# Non-pharmacological therapy for pediatric adenoid hypertrophy: a systematic review and network meta-analysis

**DOI:** 10.3389/fped.2026.1789855

**Published:** 2026-09-08

**Authors:** Fangyang Guo, Ruohan Liu, Yuxiong Zou, Xiyi Li, Mingfeng Xie, Yuening Yao, Qian Liu

**Affiliations:** 1 Integrated Chinese and Western Medicine Institute for Children Health & Drug Innovation, Discipline of Chinese and Western Integrative Medicine, Jiangxi University of Chinese Medicine, Nanchang, Jiangxi, China; 2 Suntaihe Guoyi Guoyao Clinic, Hangzhou, Zhejiang, China

**Keywords:** acupuncture, adenoid hypertrophy, moxibustion, network meta-analysis, non-pharmacological therapy

## Abstract

**Introduction:**

Adenoid hypertrophy (AH) is a common chronic inflammatory condition in children and a major cause of upper airway obstruction. Although acupuncture- and moxibustion-based therapies have been used as adjunctive treatments for pediatric AH, the relative efficacy of different modalities remains uncertain. Therefore, this network meta-analysis was conducted to compare the efficacy of different acupuncture and moxibustion interventions for pediatric AH.

**Methods:**

Eight databases were searched from their inception to May 1, 2025, for randomized controlled trials (RCTs) related to acupuncture for AH. The risk of bias was assessed using the Cochrane Risk of Bias (RoB 2.0) tool. Network meta-analysis was conducted using R 4.2.0 and Stata 14.0 software.

**Results:**

Seven kinds of acupuncture and moxibustion adjuvant intervention measures were studied, including conventional acupuncture (CA), electroacupuncture (EA), warm acupuncture (WA), conventional moxibustion (CM), thunder-fire moxibustion (TM), sandwiched moxibustion (SM), and acupoint application (AA). Adenoid/nasopharynx ratio (A/N), nasal obstruction, mouth breathing, and snore were analyzed. RoB2.0 results showed most studies were judged as having some concerns or high risk of bias, and sensitivity analysis showed low heterogeneity. CINeMA showed the confidence ratings of different treatment comparisons were judged as very low to low. The network meta-analysis demonstrated that CA + TCMs (traditional Chinese medicine) showed a clinically relevant improvement in the A/N ratio. CM + TCMs exhibited superior efficacy for nasal obstruction [MD = −1.22, 95%CI(-1.82, −0.62), *P* < 0.05] and mouth breathing [MD = −1.04, 95%CI(-1.43, −0.65), *P* < 0.05]. CA + EA + WA demonstrated advantage for snore reduction [MD = −1.00, 95%CI(-1.70, −0.30), *P* < 0.05].

**Conclusion:**

Acupuncture- and moxibustion-based adjunctive therapies may be associated with improvements in symptoms and radiological outcomes in pediatric adenoid hypertrophy. However, the certainty of the evidence ranged from very low to low, and these findings should be interpreted cautiously.

**Systematic Review Registration:**

https://www.crd.york.ac.uk/PROSPERO/home, identifier CRD420251052651.

## Introduction

Adenoid hypertrophy (AH) is a common chronic inflammatory condition in children, characterized by symptoms such as nasal obstruction, rhinorrhea, mouth breathing, and sleep disturbances (1). It is one of the leading causes of upper airway obstruction in the pediatric population. Epidemiological studies have reported a prevalence ranging from 9.9% to 34.46%, highlighting its substantial clinical and public health burden (2, 3). Current management strategies for AH primarily include adenoidectomy and pharmacological treatments, such as anti-inflammatory and anti-infective medications (4). However, concerns regarding surgical risks, postoperative recurrence, potential adverse effects of long-term medication use, and treatment-related healthcare costs have prompted increasing interest in complementary and alternative therapeutic approaches (5, 6). Therefore, identifying safe, effective, and accessible non-pharmacological interventions remains an important clinical priority.

Acupuncture and moxibustion are widely used therapeutic modalities within traditional Chinese medicine and have been applied in the management of a variety of chronic conditions (7). They are widely used globally for treating chronic intractable diseases such as AH (8, 9). These interventions are believed to regulate physiological functions through external stimulation, potentially exerting anti-inflammatory, immunomodulatory, and symptom-relieving effects while avoiding some of the adverse effects associated with pharmacological therapies (10, 11). Several clinical studies have suggested that acupuncture- and moxibustion-based interventions may improve symptoms in children with AH. However, these interventions encompass a wide range of modalities, including conventional acupuncture, electroacupuncture, warm acupuncture, conventional moxibustion, thunder-fire moxibustion, acupoint application, and other related techniques. Their comparative effectiveness remains unclear because direct head-to-head evidence is limited.

Existing studies are often constrained by relatively small sample sizes and methodological limitations, resulting in uncertainty regarding the relative benefits of different acupuncture and moxibustion approaches. To address this evidence gap, we conducted a systematic review and network meta-analysis to compare the efficacy of different acupuncture- and moxibustion-based adjunctive therapies for pediatric AH. By integrating both direct and indirect evidence, this study aims to provide a comprehensive overview of the available evidence and to identify potentially promising interventions for future clinical research and practice.

## Methods

### Protocol and registration

This study was reported according to the Preferred Reporting Items for Systematic Reviews and Meta-analysis (PRISMA) for network meta-analysis (12). The PRISMA-NMA checklist is provided in Supplementary Material S1. The International Prospective Register of Systematic Reviews (PROSPERO) and the Cochrane Library were searched for no similar studies (13). The review protocol was registered in the PROSPERO under the registration number CRD420251052651.

### Literature search strategy

We systematically searched eight English and Chinese databases, including PubMed, Web of Science, the Cochrane Library, Embase, China National Knowledge Infrastructure (CNKI), Wanfang Database, Chongqing VIP Database (CQVIP), and Chinese Biomedical Literature Service System (SinoMed), were searched through May 1, 2025, for randomized controlled trials (RCTs), without language restrictions. Detailed literature retrieval strategy see Supplementary Material S2.

### Inclusion and exclusion criteria

#### Inclusion criteria

The inclusion criteria of this study strictly follow the PICOS framework (14).

1. Population: Pediatric participants with AH, aged less than 15 years.
2. Intervention: Pediatric participants in the intervention group received acupuncture and moxibustion therapy.
3. Comparison: Pediatric participants in the control group were treated with conventional drugs.
4. Outcomes: Runny nose, snoring, and mouth breathing, and total symptom score, A/N ratio, and endoscopic grading.
5. Study design: RCTs.

#### Exclusion criteria

1. Pediatric patients with Non-AH, or adult patients.
2. Repeated publication.
3. Incorrect or incomplete data.
4. The full texts are unavailable.
5. Non-RCT design.

### Study screening

EndNote X9 software was employed to identify and remove duplicate records. Subsequently, two investigators (LRH and ZYX) independently assessed article titles and abstracts for relevance. Full texts of potentially eligible studies were then screened against the inclusion criteria. Disagreements between reviewers were resolved through consultation with a third investigator (LXY).

### Data extraction

Two investigators (LRH and ZYX) conducted data extraction independently using a structured template. In cases of disagreement, consensus was achieved through discussion with a third reviewer (LXY). When the mean value was unavailable in the full-text article, we estimated the mean and standard deviation from the median and range, interquartile range, and/or 95% confidence interval range (15).

### Risk of bias assessment

The risk of bias was assessed using the Cochrane Risk of Bias tool 2.0 (RoB 2.0) (16). Two blinded reviewers (LRH and ZYX) independently performed risk-of-bias assessments (high/low/concern), with arbitration by a third researcher (LXY) when needed.

### Data synthesis and statistical analysis

Change-from-baseline values (calculated as post-treatment mean minus baseline mean) with their standard deviations served as inputs for network meta-analysis. When standard deviations of change scores were not reported, we computed them using an assumed correlation coefficient of 0.5 applied to the following equation (17).

$$SD_{\mathrm{change}}=\sqrt{{\mathrm{SD}}_{\mathrm{baseline}}^{2}+{\mathrm{SD}}_{\mathrm{final}}^{2}-2\times r\times {\mathrm{SD}}_{\mathrm{baseline}}^{2}\times {\mathrm{SD}}_{\mathrm{final}}^{2}}$$

We conducted a network meta-analysis with 10,000 iterations using R 4.2.0 and Stata 14.0. Dichotomous outcomes were analyzed using odds ratios (ORs), while continuous outcomes were assessed via mean differences (MDs), both reported with 95% confidence intervals (CIs). Treatment rankings were evaluated using the surface under the cumulative ranking curve (SUCRA), where higher values indicate superior efficacy or safety. Heterogeneity among studies was quantified using the I² index, with conventional thresholds applied: low (*I*^2^ = 0%–49%), moderate (*I*^2^ = 50%–74%), and high (*I*^2^ = 75%–100%) heterogeneity (18).

Publication bias was assessed using adjusted funnel plots for primary outcome measures. For NMA, potential publication bias was evaluated through qualitative examination of the evidence structure. Stata 14.0 was used to generate the surface under the cumulative ranking curves for ranking intervention efficacy. Comparison-adjusted funnel plots were employed to assess potential publication bias by evaluating small-study effects. Sensitivity analyses based on risk of bias characteristics of included randomized controlled trials were performed when ≥10 outcomes were reported.

### Evidence quality evaluation

For each outcome, evidence quality was assessed using the Confidence in Network Meta-Analysis (CINeMA) online (https://cinema.ispm.unibe.ch/), a tool derived from GRADE methodology (19, 20).

## Results

### Study selection

**Figure 1:**
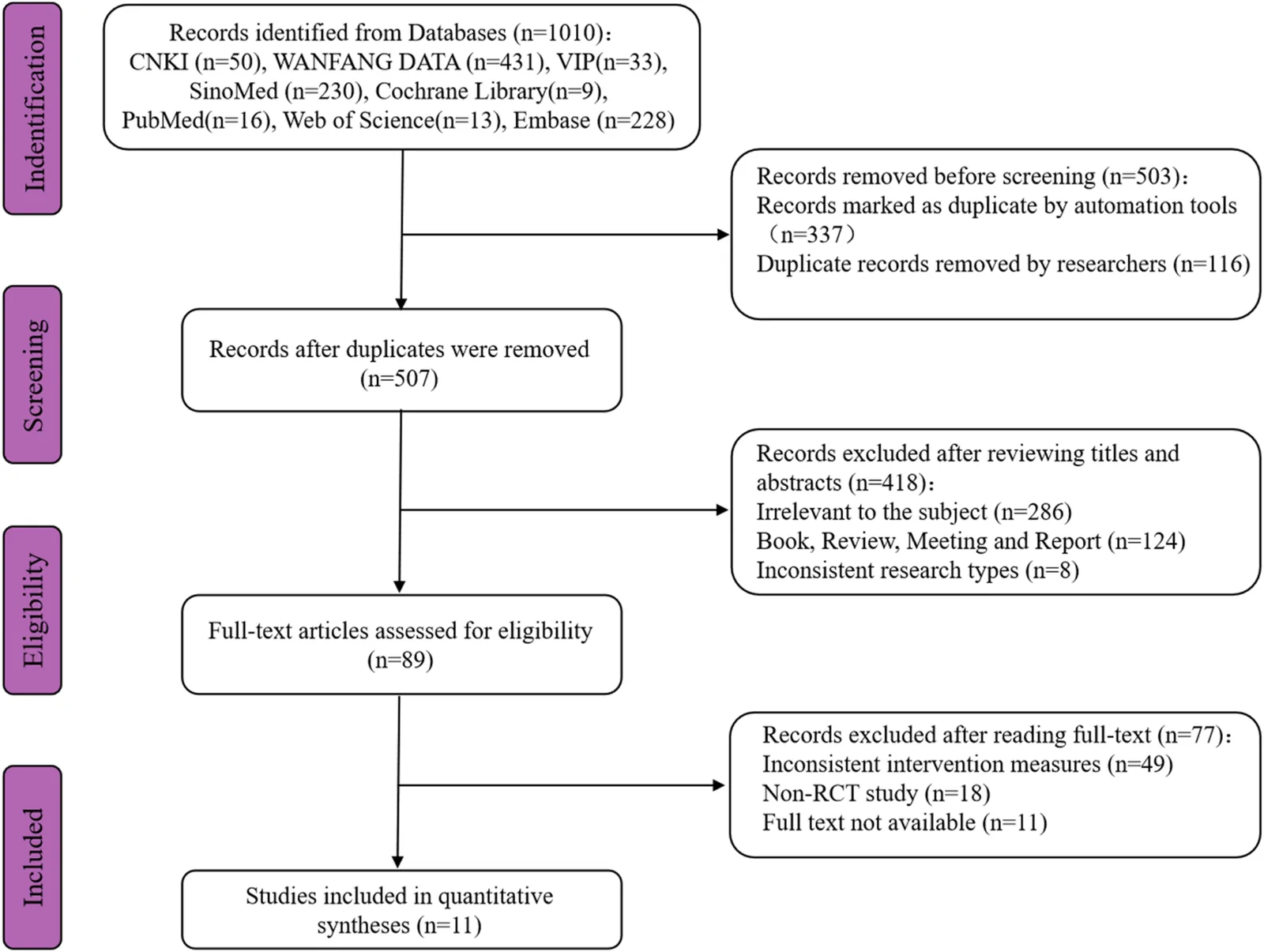
Flow chart of the systematic review process.

Initially, following the proposed search strategy, a total of 1,010 records were identified. Finally, 11 RCTs (21–31) were screened in strict accordance with the inclusion criteria. The flow chart of the selection process is presented in Figure 1.

### Study characteristics

We included 11 studies comprising 904 pediatric participants: 451 in the intervention group and 446 in the control group. The cohort demonstrated male predominance, with ages ranging from 2 to 9 years. The intervention group comprised seven modalities: conventional acupuncture (CA), electroacupuncture (EA), warm acupuncture (WA), conventional moxibustion (CM), thunder-fire moxibustion (TM), sandwiched moxibustion (SM), and acupoint application (AA). Control group participants received either Traditional Chinese Medicine formulas (TCMs) or anti-inflammatory drugs (ADs). The treatment time ranged from 14 days to 3 months. Outcome indicators included A/N ratio, Nasal Obstruction, Mouth Breathing, and Snore (shown in Table 1).

**Table 1:** Characteristics of included studies.

Study	Total	Sample		Gender (M/F)		Age (Year)		Treatment		Treatment duration	Outcomes
		IG	CG	IG	CG	IG	CG	IG	CG		
Deng (21)	60	30	30	21/9	20/10	6.3 ± 2.3	6.5 ± 2.4	CA + EA + WA	ADs	TG: 23.5 ± 14.7 days CG: 18.8 ± 13.5 days	①②③④
Liu (22)	82	41	41	18/23	21/20	7.00 ± 1.95	6.83 ± 1.92	CA + TCMs	ADs	4 weeks	①②③④
Tian (23)	80	38	37	23/15	21/16	7.03 ± 1.22	7.19 ± 1.08	CA + TCMs	ADs	14 days	①②③④
Zheng (25)	60	30	30	15/15	14/16	7.72 ± 1.72	7.68 ± 1.75	CA + TCMs	ADs	30 days	①②③④
Bi (24)	120	60	60	45/15	47/13	7.42 ± 1.33	7.43 ± 1.32	CM + TCMs	ADs	4 weeks	①②③④
Cheng (26)	60	30	28	18/12	16/14	5.26 ± 2.17	5.19 ± 2.05	CM + TCMs	TCMs	4 weeks	②④
Huang (27)	60	30	30	14/16	19/11	5.63 ± 1.63	5.70 ± 2.00	CM + TCMs	ADs	3 months	①②③④
Huo (28)	92	46	46	15/31	14/32	7.23 ± 0.45	7.26 ± 0.43	TM + TCMs	ADs	1 month	②③
Li (29)	76	38	38	25/13	27/11	5.51 ± 1.32	5.56 ± 1.45	TM + TCMs	TCMs	21 days	②④
Zhuge (30)	152	76	76	39/37	40/36	4.8 ± 1.6	5.8 ± 1.02	SM	ADs	1 month	①
Zhao (31)	62	32	30	19/13	19/11	2∼9	2∼9	AA + TCMs	ADs	1 month	②③④

CG, control group; IG, intervention group; CA, Conventional acupuncture; EA, Electroacupuncture; WA, Warm acupuncture; CM, Conventional moxibustion; TM, Thunder-fire moxibustion; SM, Sandwiched moxibustion; AA, Acupoint application; TCMs, Traditional Chinese medicine formulas; ADs, Anti-inflammatory drugs.

① A/N, ② Symptom score: nasal obstruction, ③ Symptom score: mouth breathing, ④ Symptom score: snore.

### Risk of bias within studies

**Figure 2:**
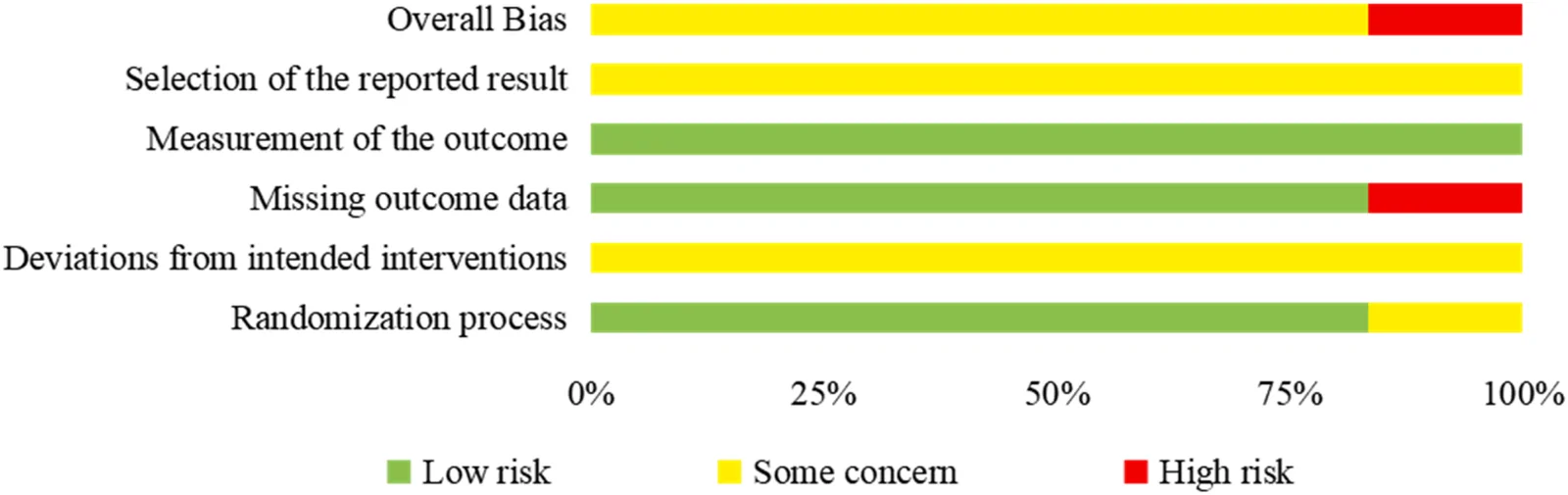
Results of the risk of bias evaluation.

All studies underwent risk of bias assessment by RoB 2.0. A study (22) failed to specify the randomization process, while two others (21, 23) exhibited high attrition risk due to missing outcome data. Consequently, the overall bias identified 18% high risk and 82% some concerns (Figure 2).

### Network meta-analysis

As the network structure contained no closed loops, and thus no potential for inconsistency, we applied a consistency model for all statistical analyses without requiring inconsistency assessment. The complete network Meta-analysis forest plot is shown in Supplementary Material S3.

### Adenoid/nasopharynx ratio

**Figure 3:**
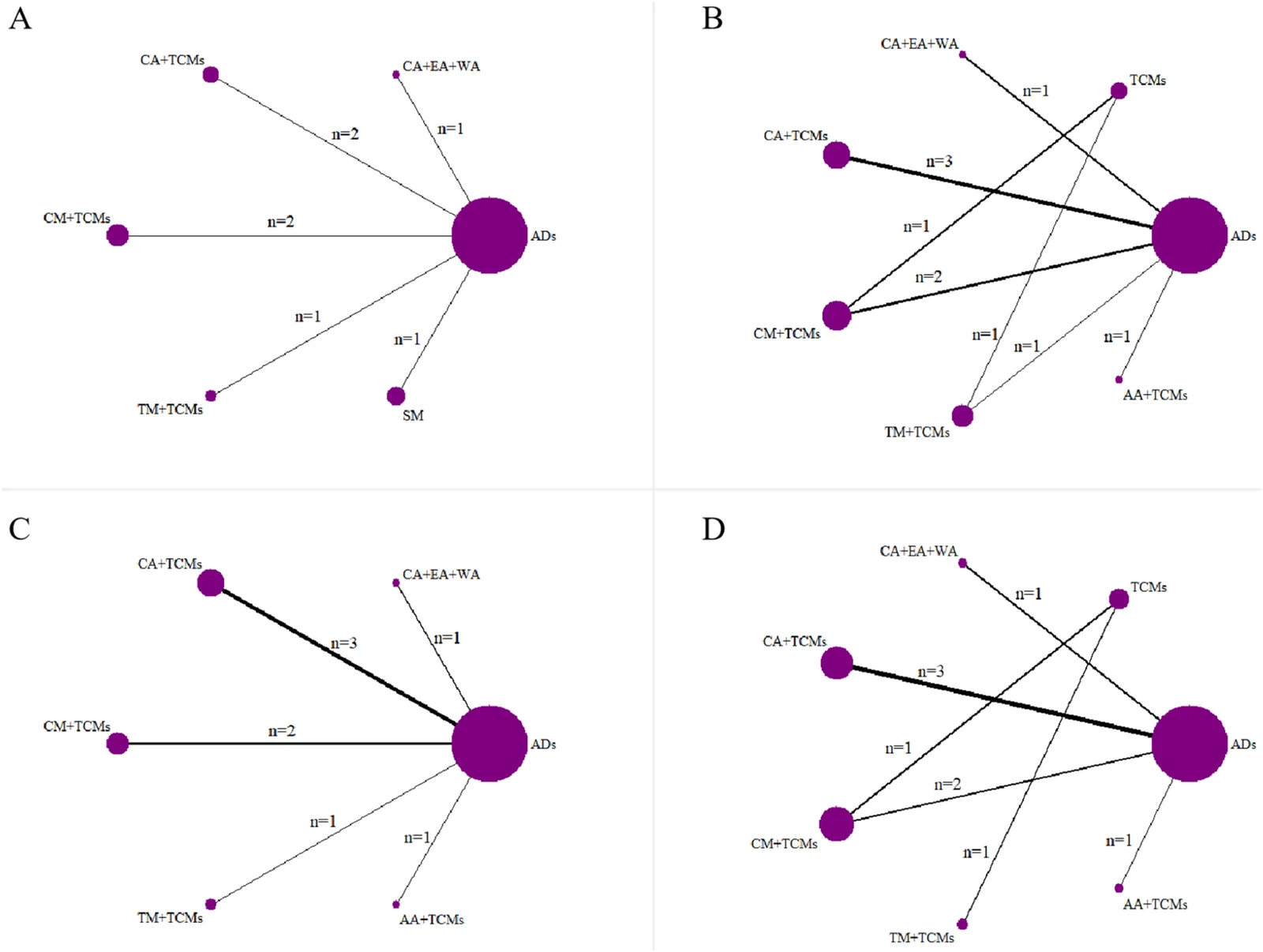
Network evidence plot. **(A)** Evidence network plot of A/N. **(B)** Evidence network plot of nasal obstruction. **(C)** Evidence network plot of mouth breathing. **(D)** Evidence network plot of snore. CA, Conventional acupuncture; EA, Electroacupuncture; WA, Warm acupuncture; CM, Conventional moxibustion; TM, Thunder-fire moxibustion; SM, Sandwiched moxibustion; AA, Acupoint application; TCMs, Traditional Chinese medicine formulas; ADs, Anti-inflammatory drugs.

**Figure 4:**
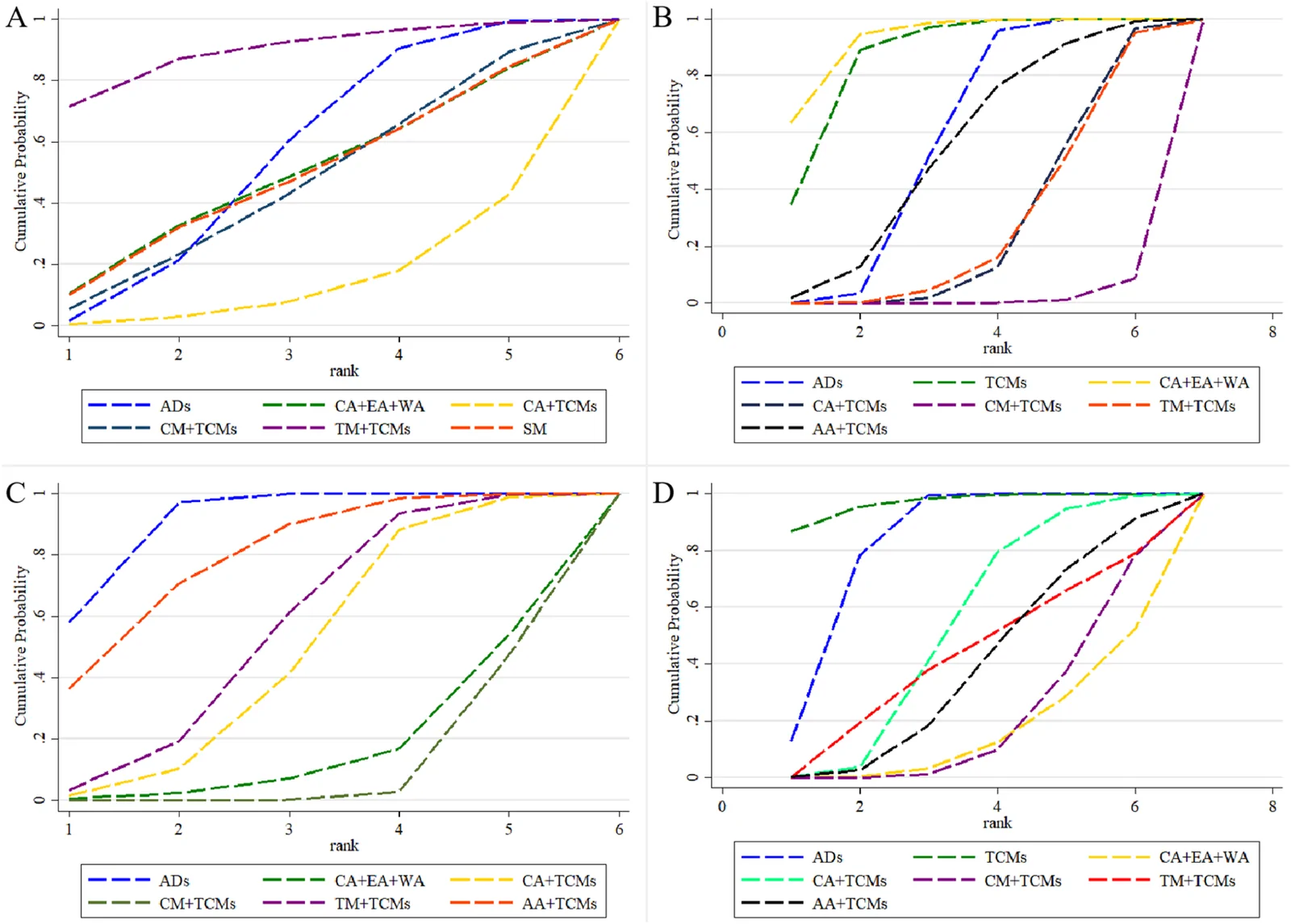
SUCRA probability ranking results. **(A)** Ranking results of A/N. **(B)** Ranking results of nasal obstruction. **(C)** Ranking results of mouth breathing. **(D)** Ranking results of snore. CA, Conventional acupuncture; EA, Electroacupuncture; WA, Warm acupuncture; CM, Conventional moxibustion; TM, Thunder-fire moxibustion; SM, Sandwiched moxibustion; AA, Acupoint application; TCMs, Traditional Chinese medicine formulas; ADs, Anti-inflammatory drugs.

A total of 7 (21, 23–25, 27, 28, 30) studies involving 619 patients were included, reporting A/N outcome measures. The network evidence plot of A/N is presented in Figure 3A. Compared to the ADs, the network meta-analysis indicated that results CA + EA + WA, CA + TCMs, CM + TCMs, and SM were better, but none of these differences were statistically significant. The league table is presented in Table 2. Based on both direct and indirect comparisons, the reduction in A/N achieved by acupuncture therapies was as follows: CA + TCMs > CM + TCMs > SM > CA + EA + WA > ADs > TM + TCMs, as shown in Figure 4A.

**Table 2:** Network meta-analysis league table of A/N.

ADs					
0.06(−0.86,0.98)	CA + EA + WA				
0.52(−0.15,1.19)	0.46(−0.68,1.60)	CA + TCMs			
0.09(−0.56,0.74)	0.03(−1.09,1.15)	−0.43(−1.36,0.50)	CM + TCMs		
−0.63 (0.31,−1.57)	−0.69(−2.00,0.62)	−1.15(−2.30,0.00)	−0.72(−1.86,0.42)	TM + TCMs	
0.06(−0.86,0.98)	0.00(−1.30,1.30)	−0.46(−1.60,0.68)	−0.03(−1.15,1.09)	0.69(−0.62,2.00)	SM

CA, Conventional acupuncture; EA, Electroacupuncture; WA, Warm acupuncture; CM, Conventional moxibustion; TM, Thunder-fire moxibustion; TCMs, Traditional Chinese medicine formulas; ADs, Anti-inflammatory drugs.

### Symptom score: nasal obstruction

A total of 10 (21–29, 31) studies involving 745 patients were included, reporting nasal obstruction outcome measures. The network evidence plot of nasal obstruction is presented in Figure 3B. Compared to the ADs, the network meta-analysis indicated that the result of CM + TCMs [MD = −1.22, 95%CI(−1.82, −0.62)] was significantly better. In addition, compared to the TCMs, the network meta-analysis indicated that the result of CA + TCMs [MD = −1.25, 95%CI(−2.25, −0.26)], CM + TCMs [MD = −1.97, 95%CI(−2.81, −1.12)], and TM + TCMs [MD = −1.28, 95%CI(−2.04, −0.51)] were significantly better. The league table is presented in Table 3. Based on both direct and indirect comparisons, the reduction of nasal obstruction achieved by acupuncture therapies was as follows: CM + TCMs > CA + TCMs > TM + TCMs > AA + TCMs > ADs > TCMs > CA + EA + WA, as shown in Figure 4B.

**Table 3:** Network meta-analysis league table of nasal obstruction.

ADs						
−0.75(−1.62,0.12)	TCMs					
−1.00(−1.95,−0.05)^a^	−0.25(−1.54,1.04)	CA + EA + WA				
0.51(−0.01,1.02)	1.25 (0.26,2.25)^a^	1.51 (0.43,2.58)^a^	CA + TCMs			
1.22 (0.62,1.82)^a^	1.97 (1.12,2.81)^a^	2.22 (1.10,3.34)^a^	0.71(−0.06,1.47)	CM + TCMs		
0.53(−0.15,1.21)	1.28 (0.51,2.04)^a^	1.53 (0.36,2.96)^a^	0.02(−0.81,0.86)	−0.69(−1.49,0.11)	TM + TCMs	
0.03(−0.77,0.83)	0.78(−0.41,1.96)	1.03(−0.21,2.27)	−0.48(−1.43,0.47)	−1.19(−2.19,−0.19)^a^	−0.50(−1.55,0.55)	AA + TCMs

a Represent statistical significance (*P* < 0.05).

CA, Conventional acupuncture; EA, Electroacupuncture; WA, Warm acupuncture; CM, Conventional moxibustion; TM, Thunder-fire moxibustion; AA, Acupoint application; TCMs, Traditional Chinese medicine formulas; ADs, Anti-inflammatory drugs.

### Symptom score: mouth breathing

A total of 8 (21–25, 27, 28, 31) studies involving 611 patients were included, reporting mouth breathing outcome measures. The network evidence plot of mouth breathing is presented in Figure 3C. Compared to the ADs, the network meta-analysis indicated that the result of CA + EA + WA [MD = −1.00, 95%CI(−1.84, −0.16)], CA + TCMs [MD = −0.46, 95%CI(−0.92, 0.00)], and CM + TCMs [MD = −1.04, 95%CI(−1.43, −0.65)] were significantly better. The league table is presented in Table 4. Based on both direct and indirect comparisons, the reduction of nasal obstruction achieved by acupuncture therapies was as follows: CM + TCMs > CA + EA + WA > CA + TCMs > TM + TCMs > AA + TCMs > ADs, as shown in Figure 4C.

**Table 4:** Network meta-analysis league table of mouth breathing.

ADs					
1.00 (0.16,1.84)^a^	CA + EA + WA				
0.46 (0.00,0.92)^a^	−0.54(−1.50,0.42)	CA + TCMs			
1.04 (0.65,1.43)^a^	0.04(−0.88,0.97)	0.58(−0.02,1.19)	CM + TCMs		
0.37(−0.07,0.81)	−0.63(−1.58,0.32)	−0.09(−0.73,0.54)	−0.67(−1.26,−0.09)	TM + TCMs	
0.09(−0.46,0.64)	−0.91(−1.91,0.09)	−0.37(−1.09,0.34)	−0.95(−1.62,−0.28)	−0.28(−0.98,0.42)	AA + TCMs

a Represent statistical significance (*P* < 0.05).

CA, Conventional acupuncture; EA, Electroacupuncture; WA, Warm acupuncture; CM, Conventional moxibustion; TM, Thunder-fire moxibustion; AA, Acupoint application; TCMs, Traditional Chinese medicine formulas; ADs, Anti-inflammatory drugs.

### Symptom score: snore

A total of 9 (21–27, 29, 31) studies involving 653 patients were included, reporting snore outcome measures. The network evidence plot of snore is presented in Figure 3D. Compared to the ADs, the network meta-analysis indicated that the result of CA + EA + WA [MD = −1.00, 95%CI(−1.70, −0.30)], CA + TCMs [MD = −0.43, 95%CI(−0.80, −0.05)], CM + TCMs [MD = −0.87, 95%CI(−1.30, −0.44)], and AA + TCMs [MD = −0.63, 95%CI(−1.21, −0.05)] were significantly better. In addition, compared to the TCMs, the network meta-analysis indicated that the result of CA + EA + WA [MD = −1.00, 95%CI(−2.85, −0.33)], CM + TCMs [MD = −1.46, 95%CI(−2.42, −0.50)], TM + TCMs [MD = −1.16, 95%CI(−1.92, −0.40)], and AA + TCMs [MD = −1.22, 95%CI(−2.42, −0.02)] were significantly better. The league table is presented in Table 5. Based on both direct and indirect comparisons, the reduction of nasal obstruction achieved by acupuncture therapies was as follows: CA + EA + WA > CM + TCMs > AA + TCMs > TM + TCMs > CA + TCMs > ADs > TCMs, as shown in Figure 4D.

**Table 5:** Network meta-analysis league table of snore.

ADs						
−0.59(−1.64,0.46)	TCMs					
1.00 (0.30,1.70)^a^	1.59 (0.33,2.85)^a^	CA + EA + WA				
0.43 (0.05,0.80)^a^	1.02(−0.11,2.14)	−0.57(−1.36,0.22)	CA + TCMs			
0.87 (0.44,1.30)^a^	1.46 (0.50,2.42)^a^	−0.13(−0.95,0.69)	0.44(−0.15,1.03)	CM + TCMs		
0.57(−0.73,1.87)	1.16 (0.40,1.92)^a^	−0.43(−1.90,1.05)	0.14(−1.22,1.51)	−0.30(−1.53,0.92)	TM + TCMs	
0.63 (0.05,1.21)^a^	1.22 (0.02,2.42)^a^	−0.37(−1.28,0.54)	0.20(−0.49,0.89)	−0.24(−0.96,0.48)	0.06(−1.37,1.48)	AA + TCMs

a Represent statistical significance *(P* *<* *0.05).*

CA, Conventional acupuncture; EA, Electroacupuncture; WA, Warm acupuncture; CM, Conventional moxibustion; TM, Thunder-fire moxibustion; AA, Acupoint application; TCMs, Traditional Chinese medicine formulas; ADs, Anti-inflammatory drugs.

### Sensitivity analysis

The Duval & Tweedie trim-and-fill procedure was applied to all four endpoints. Adjusted effect sizes remained consistent (pooled MD change: 0), indicating robustness to potential publication bias and absence of influential heterogeneity drivers (shown in Supplementary Material S4).

### Certainty assessment of evidence

Per CINeMA framework, evidence quality was compromised by major concerns in four domains: within-study bias, imprecision, heterogeneity, and incoherence across all outcomes. Therefore, the confidence rating of the treatment comparisons were evaluated as very low to low (shown in Supplementary Material S5).

### Publication bias

**Figure 5:**
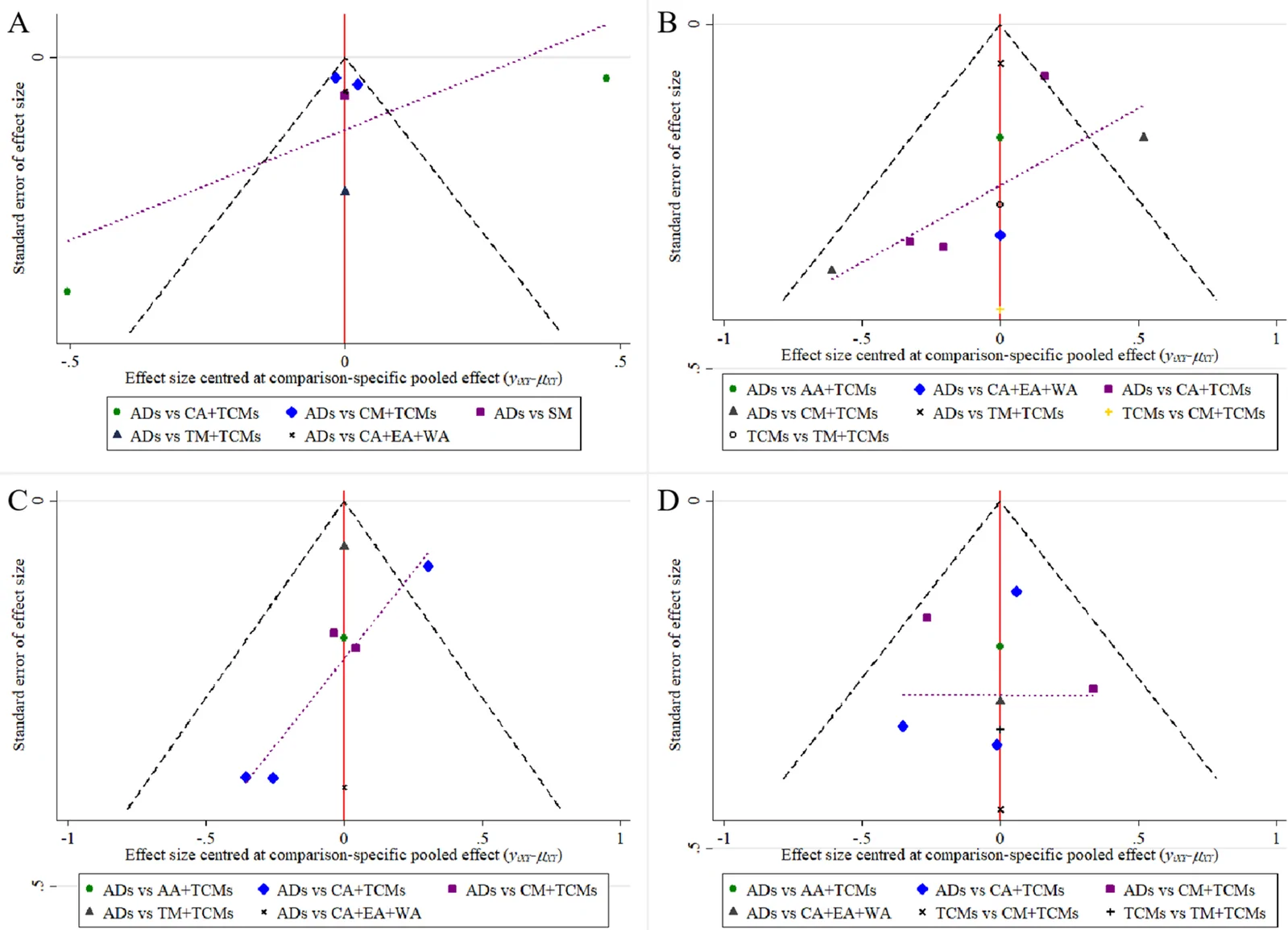
Bias detection comparison- adjusted funnel plot. **(A)** Funnel plot of A/N. **(B)** Funnel plot of nasal obstruction. **(C)** Funnel plot of mouth breathing. **(D)** Funnel plot of snore. CA, Conventional acupuncture; EA, Electroacupuncture; WA, Warm acupuncture; CM, Conventional moxibustion; TM, Thunder-fire moxibustion; SM, Sandwiched moxibustion; AA, Acupoint application; TCMs, Traditional Chinese medicine formulas; ADs, Anti-inflammatory drugs.

The comparison-adjusted funnel plots of the outcome indicators were generated using Stata 14.0 software. The results showed that the funnel plots were basically symmetrical, suggesting that there might be no publication bias in this study, as shown in Figure 5.

## Discussion

### Summary of evidence

This review compared seven acupuncture- and moxibustion-based adjunctive therapies for pediatric adenoid hypertrophy, including conventional acupuncture (CA), electroacupuncture (EA), warm acupuncture (WA), conventional moxibustion (CM), thunder-fire moxibustion (TM), sandwiched moxibustion (SM), and acupoint application (AA).

Overall, the network meta-analysis suggested that several acupuncture and moxibustion modalities may be associated with improvements in symptom scores and radiological outcomes. CA combined with traditional Chinese medicine formulations (CA + TCMs) ranked highest for improvement in the adenoid-to-nasopharynx (A/N) ratio, although no statistically significant differences were observed between interventions. CM combined with traditional Chinese medicine formulations (CM + TCMs) ranked highest for nasal obstruction and mouth-breathing outcomes according to SUCRA values, while the combined acupuncture approach (CA + EA + WA) ranked highest for snoring outcomes.

However, these findings should be interpreted with caution. According to the CINeMA assessment, the certainty of evidence ranged from very low to low across all outcomes. In addition, the network structure lacked closed loops, precluding formal assessment of inconsistency. Consequently, potential violations of the transitivity assumption underlying network meta-analysis cannot be completely excluded, and treatment rankings should be considered exploratory rather than definitive.

### Potential mechanisms

Although the precise mechanisms underlying the effects of acupuncture and moxibustion in pediatric adenoid hypertrophy remain incompletely understood, several biological pathways have been proposed. Previous studies suggest that acupuncture may modulate autonomic nervous system activity through stimulation of the sphenopalatine ganglion, potentially reducing glandular secretion, local tissue congestion, and mucosal edema, thereby contributing to improved nasal airflow and relief of upper airway obstruction (32, 33). In addition, acupuncture-mediated signaling through trigeminal afferent pathways has been associated with reduced release of pro-inflammatory neuropeptides, such as substance P and calcitonin gene-related peptide, as well as increased production of anti-inflammatory mediators, including vasoactive intestinal peptide (34, 35).

Nasal obstruction in adenoid hypertrophy is thought to result from both mechanical blockage caused by enlarged adenoid tissue and inflammatory changes within the nasopharyngeal mucosa (36). Experimental and clinical studies have suggested that acupuncture and moxibustion may improve local microcirculation and exert anti-inflammatory effects, which could contribute to reductions in nasal congestion and mucosal swelling (37). Furthermore, stimulation of perinasal acupoints, such as Yingxiang (LI20) and Yintang (EX-HN3), has been reported to increase nasal mucosal blood flow and facilitate the clearance of inflammatory mediators, potentially improving nasal patency (38, 39).

Prolonged oral respiration predisposes pediatric patients to disuse atrophy of the tensor veli palatini muscle, further reducing the oropharyngeal cross-sectional area and contributing to the development of adenoid facies (40). The characteristic snoring in children arises when accelerated airflow across the free margin of the soft palate generates a Bernoulli effect, drawing bilateral soft tissues toward the midline where they collide and vibrate (41). It has been hypothesized that acupuncture at Xiaguan (ST7) and related acupoints may influence autonomic regulation of the nasal vasculature through modulation of sphenopalatine ganglion activity, thereby reducing mucosal congestion and increasing nasal airway dimensions. Such physiological effects may partially explain the favorable symptom improvements observed in some of the included studies.

Nevertheless, the mechanistic evidence cited above is derived largely from experimental studies and conditions related to, but not specific to, pediatric adenoid hypertrophy. Therefore, these proposed mechanisms should be regarded as hypothetical and require further validation in well-designed mechanistic and clinical studies.

### Clinical significance and perspectives

In response to the WHO Global Strategy for Traditional Medicine (2025–2034) calling for evidence-based integration of complementary therapies (42), this network meta-analysis suggests that acupuncture and moxibustion modalities, as adjunctive non-pharmacological interventions for pediatric adenoid hypertrophy, may be associated with improvements in specific symptom domains. These findings provide preliminary comparative evidence that may help inform future clinical research and hypothesis generation. Consequently, this approach may reduce surgical/pharmacological dependence and alleviate familial healthcare burdens. Furthermore, the study facilitates establishing standardized protocols for integrating acupuncture parameters with conventional treatments. Further large-scale, rigorously designed multicenter trials are required before these interventions can be recommended in clinical guidelines.

### Strengths

Current systematic reviews on adenoid hypertrophy predominantly focus on evaluating the therapeutic advantages of conventional pharmacotherapies, including montelukast sodium (43), tulobuterol (44, 45), and Chinese herbal medicine (46). Additional studies have explored the utility of medical imaging in AH diagnosis (47).

This study has several methodological strengths. Firstly, stringent inclusion/exclusion criteria were implemented to enhance homogeneity across the selected studies, thereby facilitating valid comparative analyses. Secondly, our analytical approach incorporated pre-to-post treatment effect size calculations for all outcome measures, thereby improving the precision of the estimates from the network meta-analysis. No evidence of inconsistency was detected in the available analyses; however, formal inconsistency assessment was limited by the absence of closed loops.

### Limitations

This study has several limitations. First, the included studies were subject to methodological limitations, particularly inadequate reporting of allocation concealment and blinding procedures, which may have introduced a risk of bias. In addition, ethical approval was not clearly reported in several studies, raising concerns regarding research transparency and compliance. Although this network meta-analysis synthesized both direct and indirect evidence, the available evidence network was relatively sparse and lacked closed loops, limiting the ability to fully assess consistency across treatment comparisons.

Second, considerable clinical heterogeneity existed across both intervention and comparator groups. The included acupuncture- and moxibustion-related interventions varied with respect to treatment modality, stimulation intensity, treatment duration, acupoint selection, and concomitant therapies. Similarly, comparator interventions included both anti-inflammatory medications and traditional Chinese medicine formulations. Such variability may challenge the transitivity assumption underlying network meta-analysis and should be taken into account when interpreting indirect comparisons and treatment rankings.

Third, all included studies were conducted in China, which may limit the generalizability of the findings to other healthcare systems and cultural settings. Differences in acupuncture and moxibustion practice patterns, practitioner expertise, healthcare accessibility, and patient expectations may influence treatment effects. Therefore, caution is warranted when extrapolating these findings to non-Chinese populations.

Finally, most included studies focused on symptom scores and radiological indicators, such as the adenoid-to-nasopharynx (A/N) ratio. Patient-centered outcomes, including health-related quality of life, sleep quality, cognitive functioning, long-term symptom control, and avoidance of surgical intervention, were rarely reported. As a result, the clinical significance of the observed improvements remains uncertain. Future studies should incorporate validated patient-centered outcomes and long-term follow-up measures to better inform clinical decision-making.

## Conclusions

This network meta-analysis conducted a comparative effectiveness assessment of various acupuncture and moxibustion therapies as adjuvant treatments for pediatric adenoid hypertrophy. The evaluated modalities included, conventional acupuncture, electroacupuncture, warm acupuncture, conventional moxibustion, thunder-fire moxibustion, sandwiched moxibustion, and acupoint application. This network meta-analysis suggests that several acupuncture and moxibustion-based adjunctive therapies may improve symptoms associated with pediatric adenoid hypertrophy. Conventional moxibustion combined with traditional Chinese medicine ranked highly for nasal obstruction and mouth breathing, whereas combined acupuncture approaches ranked highly for snoring outcomes. However, because the certainty of evidence was low to very low and all included studies had methodological limitations, these findings should be considered exploratory and require confirmation in future high-quality randomized controlled trials.

## Data Availability

The original contributions presented in the study are included in the article/Supplementary Material, further inquiries can be directed to the corresponding author/s.
