# Supplementary material for: Non-pharmacological therapy for pediatric adenoid hypertrophy: a systematic review and network meta-analysis
Source: Front Pediatr. 2026 Sep 8;14:1789855. doi: 10.3389/fped.2026.1789855 (PMC13593853; doi:10.3389/fped.2026.1789855)
Supplement: Supplementary file 1 [file Supplementaryfile1.docx]

Supplementary Material 1

Search strategy

PubMed n=16

(("Adenoids"[Mesh] AND (Hypertrophy[Mesh] OR Hyperplasia[Mesh])) OR ("adenoid* hypertroph*"[tiab] OR "adenoidal hyperplas*"[tiab] OR "pharyngeal tonsil hypertroph*"[tiab] OR "adenoid vegetat*"[tiab] OR "adenoid enlargement"[tiab] OR ("nasal obstruction"[tiab] AND (TCM[tiab] OR "traditional Chinese medicine"[tiab])) OR "phlegm nodule*"[tiab] OR ("snoring"[tiab] OR "sleep-disordered breathing"[tiab]) OR "nasopharyngeal stenosis"[tiab])) AND (("Acupuncture Therapy"[Mesh] OR "Acupuncture, Ear"[Mesh] OR "Moxibustion"[Mesh]) OR (acupunct*[tiab] OR moxibust*[tiab] OR acupoint*[tiab] OR "auricular plaster"[tiab] OR "ear seed"[tiab] OR "ear acupressure"[tiab] OR "electroacupunct*"[tiab]))

Web of Science n=13

TS=(("adenoid* hypertroph*" OR "adenoidal hyperplas*" OR "pharyngeal tonsil hypertroph*" OR ("nasal obstruction" AND ("TCM" OR "traditional Chinese medicine")) OR "phlegm nodule*" OR "snoring" OR "sleep-disordered breathing" OR "nasopharyngeal stenosis") AND (acupunct* OR moxibust* OR acupoint* OR "auricular plaster" OR "ear seed" OR electroacupunct*))

Embase n=228

('adenoid hypertrophy'/exp OR 'adenoid hyperplasia'/exp OR 'pharyngeal tonsil hypertrophy'/exp OR 'adenoid* hypertroph*':ti,ab OR 'adenoidal hyperplas*':ti,ab OR 'pharyngeal tonsil hypertroph*':ti,ab OR ('nasal obstruction':ti,ab AND ('traditional Chinese medicine'/exp OR TCM:ti,ab)) OR 'phlegm nodule*':ti,ab OR 'snoring'/exp OR 'sleep disordered breathing'/exp OR 'nasopharyngeal stenosis'/exp) AND ('acupuncture'/exp OR 'ear acupuncture'/exp OR 'moxibustion'/exp OR 'acupressure'/exp OR acupunct*:ti,ab OR moxibust*:ti,ab OR acupoint*:ti,ab OR 'auricular plaster':ti,ab OR 'ear seed':ti,ab)

Cochrane Library n=9

(TI,AB,KW:("adenoid* hypertroph*" OR "adenoidal hyperplas*" OR "pharyngeal tonsil enlarg*" OR "adenoid vegetat*" OR "nasopharyngeal stenosis" OR ("nasal obstruction" NEAR/3 (TCM OR "traditional Chinese medicine")) OR "phlegm stagnation" OR "snoring" OR "sleep-disordered breathing" OR "lymphoid hyperplasia") AND (TI,AB,KW:("acupuncture therap*" OR "auricular acupressure" OR moxibust* OR "ear seed" OR "acupoint*" OR "embed* needle" OR "intradermal needle" OR "scalp acupuncture" OR "electroacupunct*" OR "catgut implantation" OR "cupping therapy" ))

CNKI n=50

SU=("腺样体肥大"+"咽扁桃体肥大"+"增殖体肥大"+"腺样体增生"+"腺样体肿大"+"鼻窒"+"痰核"+"鼾眠"+"窠囊"+"颃颡不开")*("针"+"灸"+"穴"+"压豆")

WANGFANG n=431

主题:("腺样体肥大" or "咽扁桃体肥大" or "增殖体肥大" or "腺样体增生" or "腺样体肿大" or "鼻窒" or "痰核" or "鼾眠" or "窠囊" or "颃颡不开")*("针" or "灸" or "穴" or "压豆")

CVIP n=33

M=("腺样体肥大" or "咽扁桃体肥大" or "增殖体肥大" or "腺样体增生" or "腺样体肿大" or "鼻窒" or "痰核" or "鼾眠" or "窠囊" or "颃颡不开")*("针" or "灸" or "穴" or "压豆")

SinoMed n=230

("腺样体肥大"[常用字段:智能] OR "咽扁桃体肥大"[常用字段:智能] OR "增殖体肥大"[常用字段:智能] OR "腺样体增生"[常用字段:智能] OR "腺样体肿大"[常用字段:智能] OR "鼻窒"[常用字段:智能] OR "痰核"[常用字段:智能] OR "鼾眠"[常用字段:智能] OR "窠囊"[常用字段:智能] OR "颃颡不开"[常用字段:智能]) AND ( "针"[常用字段:智能] OR "灸"[常用字段:智能] OR "穴"[常用字段:智能] OR "压豆"[常用字段:智能])
