# Supplementary material for: Non-pharmacological therapy for pediatric adenoid hypertrophy: a systematic review and network meta-analysis
Source: Front Pediatr. 2026 Sep 8;14:1789855. doi: 10.3389/fped.2026.1789855 (PMC13593853; doi:10.3389/fped.2026.1789855)
Supplement: Supplementary file 2 [file Supplementaryfile2.docx]

Supplementary Material 2

Fig. S3-1. The network Meta-analysis forest plot for A/N.

Fig. S3-2. The network Meta-analysis forest plot for nasal obstruction.

Fig. S3-3. The network Meta-analysis forest plot for mouth breathing.

Fig. S3-4. The network Meta-analysis forest plot for snore.
