# Supplementary material for: Non-pharmacological therapy for pediatric adenoid hypertrophy: a systematic review and network meta-analysis
Source: Front Pediatr. 2026 Sep 8;14:1789855. doi: 10.3389/fped.2026.1789855 (PMC13593853; doi:10.3389/fped.2026.1789855)
Supplement: Supplementary file 3 [file Supplementaryfile3.docx]

Supplementary Material 3

Fig. S4-1. The network Meta-analysis sensitivity analysis for A/N.

Fig. S4-2. The network Meta-analysis sensitivity analysis for nasal obstruction.

Fig. S4-3. The network Meta-analysis sensitivity analysis for mouth breathing.

Fig. S4-4. The network Meta-analysis sensitivity analysis for Snore.
