# Supplementary material for: Non-pharmacological therapy for pediatric adenoid hypertrophy: a systematic review and network meta-analysis
Source: Front Pediatr. 2026 Sep 8;14:1789855. doi: 10.3389/fped.2026.1789855 (PMC13593853; doi:10.3389/fped.2026.1789855)
Supplement: Supplementary file 4 [file Supplementaryfile4.docx]

Supplementary Material 4

CINeMA:

Table S5-1 Certainty of evidence in A/N

| Comparison | Number of studies | Within-study bias | Reporting bias | Indirectness | Imprecision | Heterogeneity | Incoherence | Confidence rating | Reason(s) for downgrading |
| --- | --- | --- | --- | --- | --- | --- | --- | --- | --- |
| Mixed evidence | | | | | | | | | |
| ADs:CA+EA+WA | 1 | Major concerns | Low risk | No concerns | Major concerns | No concerns | Major concerns | Very low | \| Within-study bias \| Imprecision \| Incoherence \| |
| ADs:CA+TCMs | 2 | Major concerns | Low risk | No concerns | No concerns | Major concerns | Major concerns | Very low | \| Within-study bias \| Heterogeneity \| Incoherence \| |
| ADs:CM+TCMs | 2 | No concerns | Low risk | No concerns | Major concerns | No concerns | Major concerns | Very low | \| Imprecision \| Incoherence \| |
| ADs:SM | 1 | No concerns | Low risk | No concerns | Major concerns | No concerns | Major concerns | Very low | \| Imprecision \| Incoherence \| |
| ADs:TM+TCMs | 1 | No concerns | Low risk | No concerns | No concerns | Major concerns | Major concerns | Very low | \| Heterogeneity \| Incoherence \| |
| Indirect evidence | | | | | | | | | |
| CA+EA+WA:CA+TCMs | 0 | Major concerns | Low risk | No concerns | Major concerns | No concerns | Major concerns | Very low | \| Within-study bias \| Imprecision \| Incoherence \| |
| CA+EA+WA:CM+TCMs | 0 | Some concerns | Low risk | No concerns | Major concerns | No concerns | Major concerns | Very low | \| Within-study bias \| Imprecision \| Incoherence \| |
| CA+EA+WA:SM | 0 | Some concerns | Low risk | No concerns | Major concerns | No concerns | Major concerns | Very low | \| Within-study bias \| Imprecision \| Incoherence \| |
| CA+EA+WA:TM+TCMs | 0 | Some concerns | Low risk | No concerns | No concerns | Major concerns | Major concerns | Very low | \| Within-study bias \| Heterogeneity \| Incoherence \| |
| CA+TCMs:CM+TCMs | 0 | Some concerns | Low risk | No concerns | Major concerns | No concerns | Major concerns | Very low | \| Within-study bias \| Imprecision \| Incoherence \| |
| CA+TCMs:SM | 0 | Some concerns | Low risk | No concerns | Major concerns | No concerns | Major concerns | Very low | \| Within-study bias \| Imprecision \| Incoherence \| |
| CA+TCMs:TM+TCMs | 0 | Some concerns | Low risk | No concerns | No concerns | Major concerns | Major concerns | Very low | \| Within-study bias \| Heterogeneity \| Incoherence \| |
| CM+TCMs:SM | 0 | No concerns | Low risk | No concerns | Major concerns | No concerns | Major concerns | Very low | \| Imprecision \| Incoherence \| |
| CM+TCMs:TM+TCMs | 0 | No concerns | Low risk | No concerns | No concerns | Major concerns | Major concerns | Very low | \| Heterogeneity \| Incoherence \| |
| SM:TM+TCMs | 0 | No concerns | Low risk | No concerns | No concerns | Major concerns | Major concerns | Very low | \| Heterogeneity \| Incoherence \| |

Table S5-2 Certainty of evidence in nasal obstruction

| Comparison | Number of studies | Within-study bias | Reporting bias | Indirectness | Imprecision | Heterogeneity | Incoherence | Confidence rating | Reason(s) for downgrading |
| --- | --- | --- | --- | --- | --- | --- | --- | --- | --- |
| Mixed evidence | | | | | | | | | |
| AA+TCMs:ADs | 1 | No concerns | Low risk | No concerns | Major concerns | No concerns | No concerns | Very low | ["Imprecision"] |
| ADs:CA+EA+WA | 1 | Major concerns | Low risk | No concerns | Major concerns | No concerns | No concerns | Very low | ["Within-study bias","Imprecision"] |
| ADs:CA+TCMs | 3 | Some concerns | Low risk | No concerns | No concerns | Major concerns | No concerns | Very low | ["Within-study bias","Heterogeneity"] |
| ADs:CM+TCMs | 2 | No concerns | Low risk | No concerns | Major concerns | No concerns | No concerns | Very low | ["Imprecision"] |
| ADs:TM+TCMs | 1 | No concerns | Low risk | No concerns | No concerns | No concerns | No concerns | High | [] |
| CM+TCMs:TCMs | 1 | No concerns | Low risk | No concerns | No concerns | No concerns | No concerns | High | [] |
| TCMs:TM+TCMs | 1 | No concerns | Low risk | No concerns | No concerns | No concerns | No concerns | High | [] |
| Indirect evidence | | | | | | | | | |
| AA+TCMs:CA+EA+WA | 0 | Some concerns | Low risk | No concerns | Major concerns | No concerns | No concerns | Very low | ["Within-study bias","Imprecision"] |
| AA+TCMs:CA+TCMs | 0 | Some concerns | Low risk | No concerns | Major concerns | No concerns | No concerns | Very low | ["Within-study bias","Imprecision"] |
| AA+TCMs:CM+TCMs | 0 | No concerns | Low risk | No concerns | Major concerns | No concerns | No concerns | Very low | ["Imprecision"] |
| AA+TCMs:TCMs | 0 | No concerns | Low risk | No concerns | No concerns | No concerns | No concerns | High | [] |
| AA+TCMs:TM+TCMs | 0 | No concerns | Low risk | No concerns | No concerns | Major concerns | No concerns | Very low | ["Heterogeneity"] |
| ADs:TCMs | 0 | No concerns | Low risk | No concerns | No concerns | No concerns | No concerns | High | [] |
| CA+EA+WA:CA+TCMs | 0 | Major concerns | Low risk | No concerns | Major concerns | No concerns | No concerns | Very low | ["Within-study bias","Imprecision"] |
| CA+EA+WA:CM+TCMs | 0 | Some concerns | Low risk | No concerns | Major concerns | No concerns | No concerns | Very low | ["Within-study bias","Imprecision"] |
| CA+EA+WA:TCMs | 0 | Some concerns | Low risk | No concerns | No concerns | No concerns | No concerns | Moderate | ["Within-study bias"] |
| CA+EA+WA:TM+TCMs | 0 | Some concerns | Low risk | No concerns | No concerns | No concerns | No concerns | Moderate | ["Within-study bias"] |
| CA+TCMs:CM+TCMs | 0 | Some concerns | Low risk | No concerns | Major concerns | No concerns | No concerns | Very low | ["Within-study bias","Imprecision"] |
| CA+TCMs:TCMs | 0 | No concerns | Low risk | No concerns | No concerns | No concerns | No concerns | High | [] |
| CA+TCMs:TM+TCMs | 0 | Some concerns | Low risk | No concerns | No concerns | No concerns | No concerns | Moderate | ["Within-study bias"] |
| CM+TCMs:TM+TCMs | 0 | No concerns | Low risk | No concerns | No concerns | No concerns | No concerns | High | [] |

Table S5-3 Certainty of evidence in mouth breathing

| Comparison | Number of studies | Within-study bias | Reporting bias | Indirectness | Imprecision | Heterogeneity | Incoherence | Confidence rating | Reason(s) for downgrading |
| --- | --- | --- | --- | --- | --- | --- | --- | --- | --- |
| Mixed evidence | | | | | | | | | |
| AA+TCMs:ADs | 1 | No concerns | Low risk | No concerns | Major concerns | No concerns | Major concerns | Very low | ["Imprecision","Incoherence"] |
| ADs:CA+EA+WA | 1 | Major concerns | Low risk | No concerns | No concerns | Major concerns | Major concerns | Very low | ["Within-study bias","Heterogeneity","Incoherence"] |
| ADs:CA+TCMs | 3 | Some concerns | Low risk | No concerns | No concerns | Major concerns | Major concerns | Very low | ["Within-study bias","Heterogeneity","Incoherence"] |
| ADs:CM+TCMs | 2 | No concerns | Low risk | No concerns | No concerns | Major concerns | Major concerns | Very low | ["Heterogeneity","Incoherence"] |
| ADs:TM+TCMs | 1 | No concerns | Low risk | No concerns | Major concerns | No concerns | Major concerns | Very low | ["Imprecision","Incoherence"] |
| Indirect evidence | | | | | | | | | |
| AA+TCMs:CA+EA+WA | 0 | Some concerns | Low risk | No concerns | Major concerns | No concerns | Major concerns | Very low | ["Within-study bias","Imprecision","Incoherence"] |
| AA+TCMs:CA+TCMs | 0 | Some concerns | Low risk | No concerns | Major concerns | No concerns | Major concerns | Very low | ["Within-study bias","Imprecision","Incoherence"] |
| AA+TCMs:CM+TCMs | 0 | No concerns | Low risk | No concerns | No concerns | Major concerns | Major concerns | Very low | ["Heterogeneity","Incoherence"] |
| AA+TCMs:TM+TCMs | 0 | No concerns | Low risk | No concerns | Major concerns | No concerns | Major concerns | Very low | ["Imprecision","Incoherence"] |
| CA+EA+WA:CA+TCMs | 0 | Major concerns | Low risk | No concerns | Major concerns | No concerns | Major concerns | Very low | ["Within-study bias","Imprecision","Incoherence"] |
| CA+EA+WA:CM+TCMs | 0 | Some concerns | Low risk | No concerns | Major concerns | No concerns | Major concerns | Very low | ["Within-study bias","Imprecision","Incoherence"] |
| CA+EA+WA:TM+TCMs | 0 | Some concerns | Low risk | No concerns | Major concerns | No concerns | Major concerns | Very low | ["Within-study bias","Imprecision","Incoherence"] |
| CA+TCMs:CM+TCMs | 0 | Some concerns | Low risk | No concerns | No concerns | Major concerns | Major concerns | Very low | ["Within-study bias","Heterogeneity","Incoherence"] |
| CA+TCMs:TM+TCMs | 0 | Some concerns | Low risk | No concerns | Major concerns | No concerns | Major concerns | Very low | ["Within-study bias","Imprecision","Incoherence"] |
| CM+TCMs:TM+TCMs | 0 | No concerns | Low risk | No concerns | No concerns | Major concerns | Major concerns | Very low | ["Heterogeneity","Incoherence"] |

Table S5-4 Certainty of evidence in snore

| Comparison | Number of studies | Within-study bias | Reporting bias | Indirectness | Imprecision | Heterogeneity | Incoherence | Confidence rating | Reason(s) for downgrading |
| --- | --- | --- | --- | --- | --- | --- | --- | --- | --- |
| Mixed evidence | | | | | | | | | |
| AA+TCMs:ADs | 1 | No concerns | Low risk | No concerns | Major concerns | No concerns | Major concerns | Very low | ["Imprecision","Incoherence"] |
| ADs:CA+EA+WA | 1 | Major concerns | Low risk | No concerns | No concerns | Major concerns | Major concerns | Very low | ["Within-study bias","Heterogeneity","Incoherence"] |
| ADs:CA+TCMs | 3 | Some concerns | Low risk | No concerns | No concerns | Major concerns | Major concerns | Very low | ["Within-study bias","Heterogeneity","Incoherence"] |
| ADs:CM+TCMs | 2 | No concerns | Low risk | No concerns | No concerns | Major concerns | Major concerns | Very low | ["Heterogeneity","Incoherence"] |
| ADs:TM+TCMs | 1 | No concerns | Low risk | No concerns | Major concerns | No concerns | Major concerns | Very low | ["Imprecision","Incoherence"] |
| CM+TCMs:TCMs | 1 | No concerns | Low risk | No concerns | No concerns | Major concerns | Major concerns | Very low | ["Heterogeneity","Incoherence"] |
| Indirect evidence | | | | | | | | | |
| AA+TCMs:CA+EA+WA | 0 | Some concerns | Low risk | No concerns | Major concerns | No concerns | Major concerns | Very low | ["Within-study bias","Imprecision","Incoherence"] |
| AA+TCMs:CA+TCMs | 0 | Some concerns | Low risk | No concerns | Major concerns | No concerns | Major concerns | Very low | ["Within-study bias","Imprecision","Incoherence"] |
| AA+TCMs:CM+TCMs | 0 | No concerns | Low risk | No concerns | No concerns | Major concerns | Major concerns | Very low | ["Heterogeneity","Incoherence"] |
| AA+TCMs:TCMs | 0 | No concerns | Low risk | No concerns | Major concerns | No concerns | Major concerns | Very low | ["Imprecision","Incoherence"] |
| AA+TCMs:TM+TCMs | 0 | No concerns | Low risk | No concerns | Major concerns | No concerns | Major concerns | Very low | ["Imprecision","Incoherence"] |
| ADs:TCMs | 0 | No concerns | Low risk | No concerns | Major concerns | No concerns | Major concerns | Very low | ["Imprecision","Incoherence"] |
| CA+EA+WA:CA+TCMs | 0 | Major concerns | Low risk | No concerns | Major concerns | No concerns | Major concerns | Very low | ["Within-study bias","Imprecision","Incoherence"] |
| CA+EA+WA:CM+TCMs | 0 | Some concerns | Low risk | No concerns | Major concerns | No concerns | Major concerns | Very low | ["Within-study bias","Imprecision","Incoherence"] |
| CA+EA+WA:TCMs | 0 | Some concerns | Low risk | No concerns | No concerns | Major concerns | Major concerns | Very low | ["Within-study bias","Heterogeneity","Incoherence"] |
| CA+EA+WA:TM+TCMs | 0 | Some concerns | Low risk | No concerns | Major concerns | No concerns | Major concerns | Very low | ["Within-study bias","Imprecision","Incoherence"] |
| CA+TCMs:CM+TCMs | 0 | Some concerns | Low risk | No concerns | No concerns | Major concerns | Major concerns | Very low | ["Within-study bias","Heterogeneity","Incoherence"] |
| CA+TCMs:TCMs | 0 | No concerns | Low risk | No concerns | Major concerns | No concerns | Major concerns | Very low | ["Imprecision","Incoherence"] |
| CA+TCMs:TM+TCMs | 0 | Some concerns | Low risk | No concerns | Major concerns | No concerns | Major concerns | Very low | ["Within-study bias","Imprecision","Incoherence"] |
| CM+TCMs:TM+TCMs | 0 | No concerns | Low risk | No concerns | No concerns | Major concerns | Major concerns | Very low | ["Heterogeneity","Incoherence"] |
| TCMs:TM+TCMs | 0 | No concerns | Low risk | No concerns | Major concerns | No concerns | Major concerns | Very low | ["Imprecision","Incoherence"] |
